# Association between benzodiazepines and suicide risk: a matched case-control study

**DOI:** 10.1186/s12888-019-2312-3

**Published:** 2019-10-26

**Authors:** Ville Cato, Fredrik Holländare, Axel Nordenskjöld, Tabita Sellin

**Affiliations:** 1 School of Medical Sciences, Örebro University, Örebro, SE-701 82 Örebro, Sweden; 2 University Health Care Research Center, Faculty of Medicine and Health, Örebro University, SE-701 82 Örebro, Sweden

**Keywords:** Suicide, Benzodiazepine, Psychopharmaceuticals, Case control

## Abstract

**Background:**

It is unclear whether benzodiazepines increase the risk of suicide. The aim of this study was to test the hypothesis that benzodiazepines are associated with an increased risk of suicide, by comparing psychopharmacological interventions between psychiatric patients who committed suicide and a group of matched controls.

**Methods:**

The case group comprised 154 psychiatric patients (101 men, 53 women; age range: 13–96 years) who had committed suicide in Örebro County, Sweden. Control psychiatric patients matched by age, sex, and main psychiatric diagnosis were selected for each case. Binary logistic regression was used to calculate odds ratios in unadjusted and adjusted models.

**Results:**

Benzodiazepine prescriptions were more common among cases than controls (65/154 [42.2%] versus 43/154 [27.9%], *p* = 0.009, odds ratio: 1.89 [95% CI: 1.17–3.03]). This association remained significant in a model adjusted for previous suicide attempts and somatic hospitalizations (odds ratio: 1.83 [95% CI: 1.06–3.14]). No statistically significant differences were seen between the groups in the use of any other subtype of psychopharmaceutical agent.

**Conclusions:**

These data indicate that benzodiazepine use may increase the risk of suicide. However, this study is limited by the potential for indication bias.

## Background

Almost one million people worldwide die due to suicide each year [1]. Globally, the annual suicide rate is estimated to be 11.4 suicides per 100,000 people, although this estimate varies between and within countries [1]. In 2012, the suicide rate was 12.1 suicides per 100,000 inhabitants in Sweden, with Örebro County having one of the highest rates at 17.7 suicides per 100,000 inhabitants [2, 3].

Mental illness is a major problem, and suicide is the second most common cause of death worldwide in adolescents and young adults [4, 5]. Risk factors for suicide include male sex, increasing age, heredity for suicide, same-sex relationships, poor childhood history (parental neglect, sexual or physical abuse), social isolation, financial difficulties, sleep disturbances, previous suicide attempts, and psychiatric diagnoses with or without somatic comorbidity [1, 2, 6–12]. The most predominant psychiatric diagnoses that increase the risk of suicide are depressive disorders [2, 8, 11, 13]. Other conditions that increase the risk of suicide include substance use disorders, anxiety disorders, eating disorders, psychotic disorders, personality disorders, and some somatic disorders (e.g., cancer and stroke) [1, 6, 7, 13–17]. In the younger population, personality disorders with or without concurrent drug abuse are common among suicide cases [2]. Individuals who die due to suicide often have multiple risk factors for suicide [1, 2].

While psychopharmaceuticals can prevent suicidal behavior [1], intervention with psychopharmaceutic drugs focuses on the underlying psychopathological syndrome or disorder, as there is no specific psychopharmacological treatment for suicidality per se [6].

The use of antidepressants (ADs) has been shown to reduce the risk of suicide among patients with mood disorders [18–20]. However, for many years the potential for ADs to increase the risk of suicide in certain groups of depressed patients has been debated. Younger patients (< 25 years) who have borderline personality disorder or do not respond to treatment with ADs may have an increased potential risk for suicidal ideation, and careful monitoring and close follow-up is necessary when prescribing these drugs [6].

Long-term treatment with lithium significantly reduces the number of suicide attempts and completed suicides in patients with bipolar disorder and recurrent depression disorders who had previously attempted suicide [21–24]. Antipsychotics are indicated for patients with a psychotic illness, e.g., schizophrenia [25]. Clozapine significantly reduces suicidality and suicide attempts in patients with schizophrenia [21]. Benzodiazepines (BZDs) are indicated for use in patients with sleep disorders, anxiety and affective disorders, delirium, alcohol withdrawal, and aggressive and violent behaviors during psychosis [26, 27]. However, there is a lack of agreement on how sedatives such as BZDs should be used and the role they should play in the treatment of psychiatric disorders [26]. In addition, drugs are often used outside their licensed indications (i.e., off-label) [26]. Another problem, particularly with regard to the use of BZDs, is that patients may exceed the recommended duration of use [28]. Short-term use (not exceeding 4 weeks) has shown a largely positive risk/benefit ratio [26, 29, 30], and some studies have shown that BZDs may even have a suicide preventative effect if taken under the right circumstances [6, 29]. BZDs lower anxiety and reduce insomnia [31], which are identified as risk factors for suicide [31, 32]. However, other studies suggest that BZDs are associated with an increased risk of suicidal behavior [13, 20, 26, 31, 33–39]. Therefore, previous studies have shown mixed results, with symptom relief and a decrease in suicide risk being evident in some cases, but an increase in the risk of suicide in other cases. Factors associated with fatal outcomes appear to vary and may be both patient-related (e.g., age, gender, somatic or psychiatric comorbidity, misuse, and overdose) and prescriber-related (long-term BZD prescribing and using BZD as the first and only intervention) [39]. The impact of BZDs on suicide risk remains uncertain, and further research is required.

Based on the previous research demonstrating BZDs to be associated with suicide [32, 36, 40, 41], we hypothesized that a prescription for BZDs would be associated with increased suicide risk in a sample of psychiatric patients. The purpose of this study was to test the hypothesis by comparing psychopharmacological interventions between psychiatric patients who committed suicide in Örebro County, Sweden, with a group of matched controls.

## Methods

### Study design and setting

This study was part of the Psychiatric Research on Suicide (PROS) project. It included all individuals who died by suicide (International Statistical Classification of Diseases, 10th Revision [ICD-10] codes X60–84) or had an uncertain cause of death (Y10–34) in Örebro County (*n* = 339) between January 1, 2007, and December 31, 2013. This case group was compared with a group of matched controls comprising individuals who did not die by suicide in the same time period. Data were gathered from the National Causes of Death Register [42], and individuals who died by suicide were considered for inclusion in the study.

**Fig. 1:**
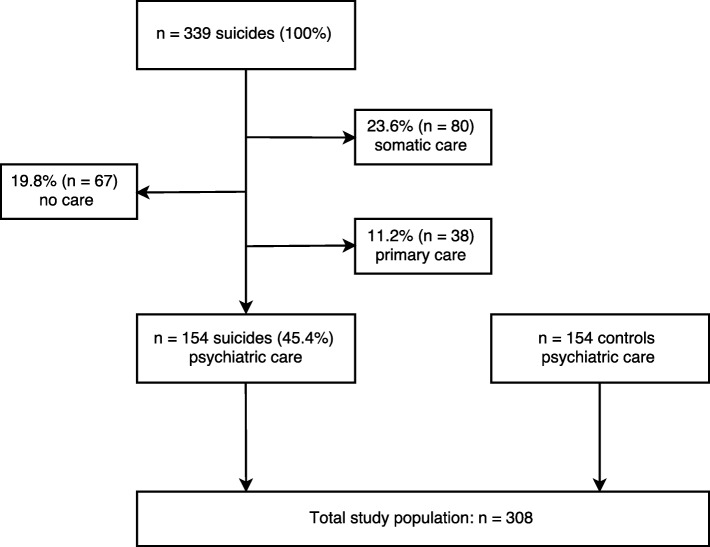
Participant flow chart. Of all individuals who committed suicide between 2007 and 2013 in Örebro County, Sweden (n = 339), 185 (54.6%) had no history of psychiatric care in the last 2 years and were excluded. The remaining 154 patients were included in this study as cases; a matched control was selected for each case

The study included only individuals who had been treated within a psychiatric outpatient or inpatient unit within the 2 years prior to suicide (*n* = 154; 45.4%). Each case was paired with a control that was matched for sex, psychiatric (F) main diagnosis (ICD-10), and age, with the most recent registered main diagnosis prior to suicide being used. The controls had received psychiatric treatment in the same year that the suicide of their matched case had occurred. The selection of the patient control group was administrated by a ‘controller function’ not included in the research team. The participant flow chart is shown in Fig. 1.

### Variables

The outcome variable was deceased by suicide (yes/no). The exposure variables considered were psychopharmacological interventions, previous suicide attempt, and previous inpatient psychiatric or somatic care in the 2 year period prior to the suicide.

All exposure variables were based on the 2 years prior to the date of suicide and were obtained from medical records. Data were gathered from Örebro County Council’s medical records, which include data on psychopharmacotherapy, diagnosis, previous suicide attempts (requiring somatic inpatient care), somatic inpatient care (previous suicide attempts excluded), and psychiatric inpatient care. The psychiatric clinics in Örebro County received an electronic pharmacy module in 2011. Prior to this, paper-based medical charts were used. Therefore, paper-based medical charts were examined from 2005 to 2011 and the electronic pharmacy model was used from 2012 to 2013 to retrieve information on prescriptions. If a psychopharmaceutic drug had been prescribed, it was categorized as follows: AD, anticonvulsant, lithium, psychostimulant, BZD, antipsychotic, or sedative (z-drugs [zopiclone, zolpidem], antihistamines).

### Statistical methods

Data were analyzed using SPSS software version 22 (IBM Corporation, Armonk, New York, USA). A two-sample independent t-test was used to compare age between the cases and controls. For the categorical variables (psychopharmaceutical prescription [yes/no], each subgroup of psychopharmaceutical prescription, and psychiatric diagnosis), data were compared across cases and controls using a Pearson chi-square test. To estimate the association between each exposure variable (psychopharmaceutical prescription, previous suicide attempt, previous inpatient psychiatric/somatic care) and suicide, the odds ratio (OR) and confidence intervals (CI), was calculated by binary logistic regression. Additionally, the matching variables were added into the unadjusted and adjusted logistic regression models together with the exposure variables, to control for possible association between characteristics used in the case-control matching process (age groups, sex, psychiatric diagnostic groups) and suicide [43]. OR, CI and *p*-value for matching variables are shown in Additional file 2. A p-value < 0.05 was considered to be statistically significant.

## Results

A comparison of the characteristics of the case and control groups (including comorbidity of alcohol/drug abuse, education level, employment status, housing, previous suicide attempts, and previous inpatient psychiatric or somatic care) is shown in Table 1. The mean age of death by suicide was approximately 47 years; however, three individuals who died by suicide were aged 18 years or younger. A total of 47 (30.5%) suicide cases died by drug poisoning (ICD-10: X60–64/Y10–12, Y14); two cases died due to BZD intoxication as the underlying cause of death, one of which had a prescription for BZDs.

**Table 1:** Characteristics of cases and controls

	All	Cases	Controls	p	Test statistic
Population	308	154	154	–	–
Male	202 (65.6)	101 (65.6)	101 (65.6)	–	–
Mean age, years	47.1 (17.0)	47.1 (17.2)	47.1 (16.8)	0.968	−0.040
Housing				0.714	0.673
Owned accommodation	130 (42.2)	67 (43.5)	63 (40.9)		
Rented	166 (53.9)	80 (51.9)	86 (55.8)		
Living with friends (*n* = 4) or lack of housing information (*n* = 8)	12 (3.9)	7 (4.5)	5 (3.2)		
Education level				0.130	4.085
Low	103 (33.4)	43 (27.9)	60 (39.0)		
Medium	146 (47.4)	79 (51.3)	67 (43.5)		
High	54 (17.5)	29 (18.8)	25 (16.2)		
Employed	95 (31.0)	49 (32.0)	46 (30.1)	0.711	0.137
Previous suicide attempt	44 (14.3)	30 (19.5)	14 (9.1)	0.009	6.788
Previous inpatient psychiatric care	155 (50.3)	80 (51.9)	75 (48.7)	0.569	0.325
Previous inpatient somatic care	80 (26.0)	50 (32.5)	30 (19.5)	0.009	6.754
Diagnosis				1.000	0.047
Mental and behavioral disorders due to psychoactive substance use (F10–19)	66 (21.4)	33 (21.4)	33 (21.4)		
Schizophrenia, schizotypal, delusional, and other non-mood psychotic disorders (F20–29)	28 (9.1)	14 (9.1)	14 (9.1)		
Bipolar disorder (F31)	14 (4.5)	7 (4.5)	7 (4.5)		
Depressive disorders (F32–34.1)	103 (33.4)	51 (33.1)	52 (33.8)		
Anxiety, dissociative, stress-related, somatoform, and other non-psychotic mental disorders (F40–48)	42 (13.6)	21 (13.6)	21 (13.6)		
Disorders of adult personality and behavior (F60–69)	22 (7.1)	11 (7.1)	11 (7.1)		
Asperger’s/ADHD (F84, F90)	6 (3.9)	3 (1.9)	3 (1.9)		
No psychiatric diagnosis	27 (8.8)	14 (9.1)	13 (8.4)		
Comorbid substance use disorder (F10–19)	29 (9.4)	15 (9.7)	14 (9.1)	0.845	0.038

Data are presented as n (%) or mean (standard deviation). *P* values were obtained from a chi-square test (df = 1 [education level, df = 2; diagnosis, df = 7]) for categorical variables or an independent samples t-test for continuous variables, comparing cases and controls. Diagnoses were F01–99 (mental, behavioral, and neurodevelopmental disorders) in the International Statistical Classification of Diseases, 10th Revision classifications (ICD-10)

No psychiatric diagnosis: patients encountering psychiatric care without receiving any main psychiatric diagnosis

Number of patients with missing data: educational level, *n* = 5; employment, *n* = 2; housing, n = 8

ADHD, attention deficit hyperactivity disorder

### Association between exposure variables and suicide

The occurrence of psychopharmaceutic prescriptions was common in the study population and did not differ between cases (88.3%) and controls (90.2%). The three most common psychopharmaceutic drug prescriptions in the total sample were ADs (68.8%), sedatives (60.7%), and BZDs (35.1%). Only the prescription of BZDs differed significantly between the case and control groups (Table 2, Additional file 2). Two other exposure variables (previous suicide attempts and somatic inpatient care) showed a significant association with suicide in unadjusted analyses. In the adjusted model, BZD prescription and previous suicide attempt remained significantly associated with an increased risk of suicide.

**Table 2:** Association between exposure variables and suicide risk

Variables	Cases n (%)	Controls n (%)	Unadjusted		Adjusted^a,b^	
			OR (95% CI)	p	OR (95% CI)	p
Psychopharmaceutical prescription						
Benzodiazepine	65 (42.2)	43 (27.9)	**1.89 (1.17–3.03)**	**0.009**	**1.83 (1.06–3.14)**	**0.029**
Antidepressant	105 (68.2)	107 (69.5)	0.94 (0.58–1.53)	0.806	0.88 (0.50–1.56)	0.654
Anticonvulsant	36 (23.4)	37 (24.0)	0.97 (0.57–1.63)	0.893	0.89 (0.50–1.60)	0.697
Lithium	6 (3.9)	8 (5.2)	0.74 (0.25–2.19)	0.586	0.77 (0.20–2.90)	0.696
Psychostimulant	6 (3.9)	6 (3.9)	1.00 (0.32–3.17)	1.000	0.96 (0.24–3.86)	0.950
Antipsychotic	37 (24.0)	39 (25.3)	0.93 (0.56–1.57)	0.792	0.83 (044–1.58)	0.572
Sedative	98 (63.6)	89 (57.8)	1.28 (0.81–2.02)	0.294	1.29 (077–2.18)	0.332
Previous suicide attempt	30 (19.5)	14 (9.1)	**2.42 (1.28–4.77)**	**0.011**	**2.12 (1.01–4.44)**	**0.047**
Previous inpatient psychiatric care	80 (51.9)	75 (48.7)	0.88 (0.56–1.37)	0.569	1.07 (0.64–1.79)	0.798
Previous inpatient somatic care	50 (32.5)	30 (19.5)	**1.99 (1.18–3.35)**	**0.010**	1.69 (0.95–3.01)	0.074

^a^ Adjusted for all variables listed in the table

^b^ Adjusted for the matching variables: age groups, sex and diagnostic groups. Data shown in Additional file 2

CI, confidence interval; OR, odds ratio, significant associations in bold

### Subgroup analyses stratified by sex, diagnosis, and age

The distribution of BZD prescriptions in cases and controls stratified according to different characteristics showed a significant association between BZD prescription and suicide in women (χ^2^: 8.69, df: 1, *p* = 0.003), patients with psychotic disorders (F20–29, χ^2^: 5.25, df: 1, *p* = 0.022), patients with personality disorders (F60–69, χ^2^: 5.24, df: 1, p = 0.022), patients without a previous suicide attempt (χ^2^: 5.75, df: 1, *p* = 0.017), and patients in the age groups 30–49 years (χ^2^: 4.02, df: 1, *p* = 0.045) and 50–69 years (χ^2^: 4.67, df: 1, *p* = 0.031). Data are shown in Additional file 1.

## Discussion

The study found that BZDs were prescribed to a higher proportion of psychiatric patients who died due to suicide than controls. Therefore, our results support the hypothesis that BZDs are associated with suicide. Almost 90% of patients in both groups had a psychopharmaceutical prescription, but BZDs were prescribed to only a minority of patients in both the case and control groups. There was no significant difference in the frequency of prescription regarding other subtypes of psychopharmaceuticals between the groups.

Previous inpatient somatic care showed a significant association with suicide in the unadjusted, but not the adjusted, model. After adjusting for all variables, BZDs and previous suicide attempts were seen to be associated with suicide in this population. A previous suicide attempt is known to be one of the major risk factors for suicide [1, 2, 6].

Our results are in agreement with those presented in previous studies. Neuner et al. [40] and Taiminen et al. [41] conducted case-control studies comparing inpatients who died due to suicide during hospitalization with matched controls. Both studies found that the rate of BZD prescription was higher in the suicide group than in the control group (53% versus 38%, respectively [*p* = 0.027], in the Neuner et al. study; 72% versus 44% [*p* < 0.05] in the Taiminen et al. study). In the present study, we found the rate of BZD prescription to be similar to that seen in the previous studies (42% in cases versus 28% in controls, *p* = 0.009). Tiihonen et al. studied over 2000 patients with schizophrenia and reported that BZD use was associated with an increased risk of suicide (hazard ratio: 3.83; 95% CI: 1.45–10.12) [36].

There are two possible interpretations of the study results. The association between BZDs and suicide may arise because BZDs are prescribed to individuals who are already at increased risk of suicide as they exhibit the symptoms of anxiety and insomnia (indication bias) [44]. Alternatively, it could be considered that BZDs may causally increase the risk of suicide. Due to the study design, it is not possible to determine whether suicides in patients with BZDs were activated by an increase in aggression and impairment of behavioral inhibition, or as a result of withdrawal from BZDs, resulting in rebound of symptoms of anxiety or insomnia, as suggested by Dodds et al. [32]. However, we can conclude that, despite the suggestion of previous investigators [31, 33, 45–47], suicide as a result of BZD overdose was uncommon in our sample, with only two cases recorded.

### Study strengths and limitations

The study population comprised all individuals in the county with psychiatric contact 2 years prior to suicide who died during the study period. A strength of the study is the individual matching procedure, which identified one similar control per suicide case. The distribution of psychiatric diagnoses, mean age, and sex was similar between the two groups, indicating that there were no notable differences between cases and controls. A limitation regarding the matching of psychiatric diagnosis along with age and sex is that it was only possible to find one control per suicide case and it was not always possible to control for symptom severity. However, in the majority of cases it was possible to match cases with a control with the same third position number, reflecting the severity of the diagnosis. This also applies to comorbidities, such as concurrent anxiety or personality disorders. However, previous inpatient psychiatric care could be seen as a marker of disease severity and was balanced between the two groups, suggesting that the matching process was successful.

A limitation of the study is the potential for indication bias. BZD prescription may have been influenced by anxiety symptoms, which may also influence suicide risk. Data regarding psychopharmaceutical prescriptions were obtained from medical records. While the suicide risk associated with BZDs is thought to be dose-dependent [32], it was not possible to analyze dosage in the current study and no information was available to demonstrate whether the individual retrieved the prescribed drug or not.

Another limitation of the current study is that some patients treated prior to 2011 had only written medical records regarding pharmacotherapy. If documentation was poor, it may have influenced the results by leading to an underestimation of the rate of psychopharmaceutical prescriptions. However, the risk of differential bias between cases and controls is low. There is also the possibility of other uncontrolled risk factors associated with suicide in this study, which may have affected the results, e.g., homelessness and other socioeconomic problems [48]. However, the distribution of some important indicators of these risk factors is described in Table 1, and no significant differences were seen between the groups.

## Conclusions

The findings of this study suggest that the prescription of BZDs should be considered with caution, particularly in patients exhibiting signs of an ongoing suicidal process. BZDs may be associated with an increased risk of suicide, and possible risks and potential benefits must be assessed for each individual patient. Additional studies are required to determine any causal mechanisms and to identify additional explanations for the association. Intervention studies, such as randomized controlled trials, would be welcome, but very large sample sizes would be required as suicide is a rare event.

## Supplementary information

**Additional file 1..** An overview of benzodiazepine prescriptions. Data obtained from a two-sample chi-square test for categorical variables (df = 1), comparing cases and controls regarding benzodiazepine prescriptions. (DOCX 23 kb)

**Additional file 2..** Continuation/extension of Table 2: Association between exposure variables, including matching variables in the case-control design, and suicide risk. Data obtained from unadjusted and adjusted logistic regression. (DOCX 48 kb)

## Data Availability

The data are protected by the Public Access to Information and Secrecy Act, but can be made available for researchers from the corresponding author Axel Nordenskjöld upon request.

## Abbreviations

AD: Antidepressant
BZD: Benzodiazepine
Df: Degrees of freedom
ICD-10: International Statistical Classification of Diseases, 10th Revision
OR: Odds ratio
