# Supplementary material for: Association between benzodiazepines and suicide risk: a matched case-control study
Source: BMC Psychiatry. 2019 Oct 26;19:317. doi: 10.1186/s12888-019-2312-3 (PMC6815437; doi:10.1186/s12888-019-2312-3)
Supplement: Supplementary file 1 — Additional file 1.. An overview of benzodiazepine prescriptions. Data obtained from a two-sample chi-square test for categorical variables (df = 1), comparing cases and controls regarding benzodiazepine prescriptions. (DOCX 23 kb) [file 12888_2019_2312_MOESM1_ESM.docx]

**Additional file 1.** An overview of benzodiazepine prescriptions.

|  | **Cases** | | **Controls** | | **p** |
| --- | --- | --- | --- | --- | --- |
|  | **BZD** | **No BZD** | **BZD** | **No BZD** |  |
| **Sex** |  |  |  |  |  |
| Male | 35 (34.7) | 66 (65.3) | 28 (27.7) | 73 (72.3) | 0.288 |
| Female | 30 (56.6) | 23 (43.4) | 15 (28.3) | 38 (71.7) | 0.003 |
| **Diagnosis** |  |  |  |  |  |
| Mental and behavioral disorders due to psychoactive substance use (F10–19) | 9 (27.3) | 24 (72.7) | 7 (21.2) | 26 (78.8) | 0.566 |
| Schizophrenia, schizotypal, delusional, and other non-mood psychotic disorders (F20–29) | 9 (64.3) | 5 (35.7) | 3 (21.4) | 11 (78.6) | 0.022 |
| Bipolar disorder (F31) | 4 (57.1) | 3 (42.9) | 1 (14.3) | 6 (85.7) | 0.094 |
| Depressive disorders (F32–34.1) | 20 (39.2) | 31 (60.8) | 18 (34.6) | 34 (65.4) | 0.629 |
| Anxiety, dissociative, stress-related, somatoform, and other non-psychotic mental disorders (F40–48) | 12 (57.1) | 9 (42.9) | 8 (38.1) | 13 (61.9) | 0.217 |
| Disorders of adult personality and behavior (F60–69) | 6 (54.5) | 5 (45.5) | 1 (9.1) | 10 (90.9) | 0.022 |
| Asperger’s/ADHD (F84, F90) | 0 (0.0) | 3 (100) | 1 (33.3) | 2 (66.7) | 0.273 |
| No psychiatric diagnosis | 5 (35.7) | 9 (64.3) | 4 (30.8) | 9 (69.2) | 0.785 |
| **Previous suicide attempt** |  |  |  |  |  |
| Yes | 15 (50.0) | 15 (50.0) | 6 (42.9) | 8 (57.1) | 0.659 |
| No | 50 (40.3) | 74 (59.7) | 37 (26.4) | 103 (73.6) | 0.017 |
| **Age** |  |  |  |  |  |
| 13–29 years | 6 (18.2) | 27 (81.8) | 6 (19.4) | 25 (80.6) | 0.904 |
| 30–49 years | 16 (34.0) | 31 (66.0) | 8 (16.3) | 41 (83.7) | 0.045 |
| 50–69 years | 35 (56.5) | 27 (43.5) | 23 (37.1) | 39 (62.9) | 0.031 |
| 70–96 years | 8 (66.7) | 4 (33.3) | 6 (50.0) | 6 (50.0) | 0.408 |

Data are presented as n (%). P values were obtained from a two-sample chi-square test for categorical variables (df = 1), comparing cases and controls regarding benzodiazepine prescriptions. Diagnoses were F01–99 (mental, behavioral, and neurodevelopmental disorders) in the International Statistical Classification of Diseases, 10^th^ Revision classifications (ICD-10).

ADHD, attention deficit hyperactivity disorder; BZD, benzodiazepine.
