# Supplementary material for: Association between benzodiazepines and suicide risk: a matched case-control study
Source: BMC Psychiatry. 2019 Oct 26;19:317. doi: 10.1186/s12888-019-2312-3 (PMC6815437; doi:10.1186/s12888-019-2312-3)
Supplement: Supplementary file 2 — Additional file 2.. Continuation/extension of Table 2: Association between exposure variables, including matching variables in the case-control design, and suicide risk. Data obtained from unadjusted and adjusted logistic regression. (DOCX 48 kb) [file 12888_2019_2312_MOESM2_ESM.docx]

**Additional file 2.** Continuation/extension of table 2: Association between exposure variables, including matching variables in the case-control design, and suicide risk.

| Variables | Cases n (%) | | Controls n (%) | | Unadjusted | | | | Adjusted^a^ | | | |  |
| --- | --- | --- | --- | --- | --- | --- | --- | --- | --- | --- | --- | --- | --- |
|  |  |  |  |  | **OR (95% CI)** | | **p** | | **OR (95% CI)** | | **p** | |  |
| Age | | | | | | | | | | | | |  |
| 13-29 years | | 33 (19.5) | | 31 (20.1) | | 1.07 (0.42–2.72) | | 0.896 | | 1.55 (0.54–4.45) | | 0.421 | |
| 30-49 years | | 47 (30.5) | | 49 (31.8) | | 0.96 (0.39– 2.35) | | 0.93 | | 1.42 (0.53–3.82) | | 0.484 | |
| 50-69 years | | 62 (40.3) | | 62(40.3) | | 1.00 (0.42–3.40) | | 1.00 | | 1.15 (0.45–2.93) | | 0.769 | |
| 70-96 years | | 12 (7.8) | | 12 (7.8) | | reference | |  | | reference | |  | |
| Sex (Male) | 101 (65.6) | | 101 (65.6) | | 1.00 (0.63 –1.60) | | 1.00 | | 1.06 (0.63–1.79) | | 0.825 | |  |
| Diagnostic groups |  | |  | |  | |  | |  | |  | |  |
| Mental and behavioral disorders due to psychoactive substance use (F10–19) | 33 (21.4) | | 33 (21.4) | | 1.00 (0.19–5.32) | | 1.00 | | 0.67 (0.095–4.77) | | 0.692 | |  |
| Schizophrenia, schizotypal, delusional, and other non-mood psychotic disorders (F20–29) | 14 (9.1) | | 14 (9.1) | | 1.00 (0.17–5.83) | | 1.00 | | 0.84 ( 0.10–6.79) | | 0.870 | |  |
| Bipolar disorder (F31) | 7 (4.5) | | 7 (4.5) | | 1.00 (0.15–6.77) | | 1.00 | | 0.98 (0.10–9.77) | | 0.984 | |  |
| Depressive disorders (F32–34.1) | 51 (33.1) | | 52 (33.8) | | 0.98 (0.19–5.09) | | 0.98 | | 0.77 (0.11–5.38) | | 0.791 | |  |
| Anxiety, dissociative, stress-related, somatoform, and other non-psychotic mental disorders (F40–48) | 21 (13.6) | | 21 (13.6) | | 1.00 (0.18–5.53) | | 1.00 | | 0.69 (0.092–5.10) | | 0.713 | |  |
| Disorders of adult personality and behavior (F60–69) | 11 (7.1) | | 11 (7.1) | | 1.00 (0.16–6.08) | | 1.00 | | 0.64 (0.08–5.16) | | 0.678 | |  |
| Asperger’s/ADHD (F84, F90) | 3 (1.9) | | 3 (1.9) | | reference | |  | | reference | |  | |  |
| No psychiatric diagnosis | 14 (9.1) | | 13 (8.4) | | 1.08 (0.18–6.32) | | 0.935 | | 0.87 (0.11–6.70) | | 0.892 | |  |
| Psychopharmaceutical prescription |  | |  | |  | |  | |  | |  | |  |
| Benzodiazepine | 65 (42.2) | | 43 (27.9) | | **1.89 (1.17–3.03)** | | **0.009** | | **1.83 (1.06–3.14)** | | **0.029** | |  |
| Antidepressant | 105 (68.2) | | 107 (69.5) | | 0.94 (0.58–1.53) | | 0.806 | | 0.88 (0.50–1.56) | | 0.654 | |  |
| Anticonvulsant | 36 (23.4) | | 37 (24.0) | | 0.97 (0.57–1.63) | | 0.893 | | 0.89 (0.50–1.60) | | 0.697 | |  |
| Lithium | 6 (3.9) | | 8 (5.2) | | 0.74 (0.25–2.19) | | 0.586 | | 0.77 (0.20-2.90) | | 0.696 | |  |
| Psychostimulant | 6 (3.9) | | 6 (3.9) | | 1.00 (0.32–3.17) | | 1.000 | | 0.96 (0.24–3.86) | | 0.950 | |  |
| Antipsychotic | 37 (24.0) | | 39 (25.3) | | 0.93 (0.56–1.57) | | 0.792 | | 0.83 (044–1.58) | | 0.572 | |  |
| Sedative | 98 (63.6) | | 89 (57.8) | | 1.28 (0.81–2.02) | | 0.294 | | 1.29 (077–2.18) | | 0.332 | |  |
| Previous suicide attempt | 30 (19.5) | | 14 (9.1) | | **2.42 (1.28–4.77)** | | **0.011** | | **2.12 (1.01–4.44)** | | **0.047** | |  |
| Previous inpatient psychiatric care | 80 (51.9) | | 75 (48.7) | | 0.88 (0.56–1.37) | | 0.569 | | 1.07 (0.64–1.79) | | 0.798 | |  |
| Previous inpatient somatic care | 50 (32.5) | | 30 (19.5) | | **1.99 (1.18–3.35)** | | **0.010** | | 1.69 (0.95–3.01) | | 0.074 | |  |

^a^ Adjusted for all variables in the table
CI, confidence interval; OR, odds ratio. Significant associations in bold.

Diagnoses were F01–99 (mental, behavioral, and neurodevelopmental disorders) in the International Statistical Classification of Diseases, 10^th^ Revision classifications (ICD-10). No psychiatric diagnosis: patients encountering psychiatric care without receiving any main psychiatric diagnosis. ADHD: attention deficit hyperactivity disorder
